# Consumers in a Sustainable Food Supply Chain (COSUS): Understanding Consumer Behavior to Encourage Food Waste Reduction

**DOI:** 10.3390/foods6120104

**Published:** 2017-11-27

**Authors:** Harald Rohm, Marije Oostindjer, Jessica Aschemann-Witzel, Claudia Symmank, Valérie L. Almli, Ilona E. de Hooge, Anne Normann, Kostas Karantininis

**Affiliations:** 1 Chair of Food Engineering, Technische Universität Dresden, 01062 Dresden, Germany; claudia.symmank@tu-dresden.de; 2 Department of Chemistry, Biotechnology and Food Science, Norwegian University of Life Sciences, NO-1430 Ås, Norway; marije.oostinder@nmbu.no; 3 MAPP—Research Centre on Value Creation in the Food Sector, Aarhus University, 8210 Aarhus, Denmark; jeaw@mgmt.au.dk; 4 Nofima AS, Postboks 210, NO-1431 Ås, Norway; valerie.lengard.almli@nofima.no; 5 Department of Marketing and Consumer Behaviour, Wageningen University and Research, 6700 EW Wageningen, The Netherlands; ilona.dehooge@wur.nl; 6 RISE—The Swedish Research Institute—Bioscience and Materials, SE 402 29 Göteborg, Sweden; anne.normann@ri.se; 7 Department of Economics, Swedish University of Agricultural Sciences, 750 07 Uppsala, Sweden; karantininis.konstantinos@slu.se

**Keywords:** food waste, suboptimal food, consumer perception, choice behavior

## Abstract

Consumers are directly and indirectly responsible for a significant fraction of food waste which, for a large part, could be avoided if they were willing to accept food that is suboptimal, i.e., food that deviates in sensory characteristics (odd shape, discoloration), or that has a best-before date which is approaching or has passed, but that is still perfectly fine to eat. The choice to accept or discard suboptimal food is taken either before or after purchase (hence, in the retail store or in the household). The aim of the European research project COSUS (Consumers in a sustainable food supply chain) was to increase consumer acceptance of suboptimal food, before and after purchase, by implementing targeted strategies that are based on consumer insights, and that are feasible for and acceptable by the food sector. To reach this aim, different methodological approaches were applied to analyze this issue, to experiment with different aspects, and to test the resulting interventions. Each of these approaches was undertaken by competent consortium partners from Denmark, Germany, Norway, Sweden and The Netherlands. The project finally provides validated strategies to promote the distribution and consumption of suboptimal foods, thereby improving resource efficiency in the food chain and contributing to a more sustainable food supply.

## 1. Introduction

A key element contributing to unsustainable food production and consumption is food waste. Approximately one third of all food is wasted at some point along the food value chain [1]. With this amount of waste it is difficult to support the growing human population, estimated to reach 9 billion by 2050 [1,2,3]. Due to this expected population rise and the accompanying worry for food shortage, it is important to maximize the use of the produced food. This means that all food that is or can be made suitable for human consumption should indeed be used for this purpose, and neither be discarded nor used as lower-value secondary resource (e.g., as animal feed, or as energy source) [4].

A devaluation of foods may be triggered at all levels and by all actors in the food supply chain, including the consumer. Even when producers use technological innovations to reduce food waste (e.g., by developing new products from left raw materials, or by introducing new packaging technologies for ensuring longer shelf-life), these can only be successful when they are accepted by the end user who decides which food is actually bought in store instead of being discarded, and who also decides what food is consumed instead of being thrown away in the household. 

A significant part of the waste occurring through the entire farm-to-fork value chain may be directly or indirectly caused by the unwillingness to buy and consume products that are visually suboptimal. These visually suboptimal foods deviate from consumption standards, either due to odd shape or color, because of other imperfections, or because of being close to the best-before date, without a deteriorating impact on eating quality [3,5]. As a consequence, these products that are in fact suitable for human consumption are neither bought nor consumed but discarded or, in the best way, processed into other (lower value) products. Producers discard up to 30% of fruit and vegetables because of aesthetic imperfections, and food that has gone past the best-before date accounts for 55% of consumer food waste; 75% of this waste may be avoidable [6,7]. To encourage sustainable food choices by consumers, it is necessary to gain an in-depth understanding of internal (e.g., attitudes, motives, sensory perceptions) and external (e.g., social influence, information provision, food waste initiatives) factors that foster or hamper sustainable food choice, in particular, the choice for visually suboptimal food products.

The Consumers in a sustainable food supply chain (COSUS) project, executed 2014–2017 in the Sustainable Food Consumption and Production (SUSFOOD) program (www.susfood-db-era.net) involved six research partners from five countries: Denmark, Germany, Norway, Sweden, and The Netherlands. The project, coordinated by author M.O., aimed at analyzing and increasing consumer acceptance of suboptimal food by:

- Understanding the barriers and facilitators for acceptance of visually suboptimal foods both before and after purchase through the consumer;
- Designing and testing strategies to promote the consumption of visually suboptimal foods; and
- Investigating how winning strategies can be successfully implemented in the food chain.

**Figure 1:**
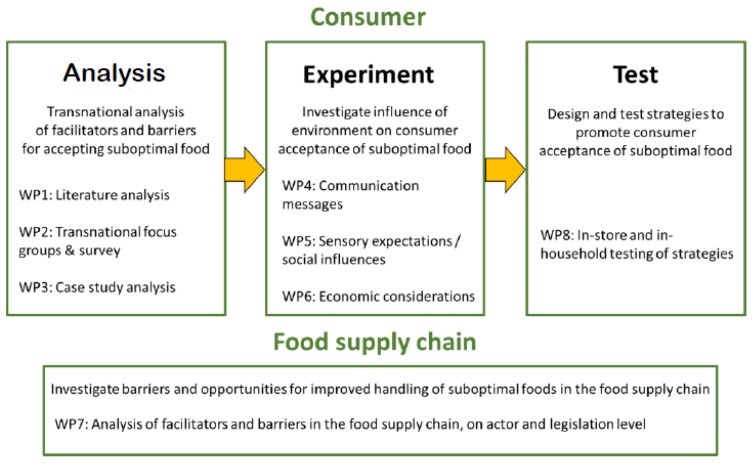
Outline of the Consumers in a sustainable food supply chain (COSUS) project. WP, workpackage conducted in the project.

This project report gives a concise summary of the results of the empirical work that has been performed under COSUS (https://cosus.nmbu.no). Different methodological approaches were applied to analyze the issue, to experiment with different aspects, and to test the resulting interventions (Figure 1). At the end, this project report draws final conclusions from the outcomes.

## 2. Suboptimal Foods and Food Waste

### 2.1. Product-Related Factors

A large share of food wasted by the consumer is avoidable: Consumers throw away food that is in fact fine to eat, but that does not meet expectations in terms of how the food looks or because the best-before date is approaching or has passed [3]. To encourage consumers to accept foods that are allegedly not optimal in sensory characteristics (shape, color) or because of a close best-before date, a first step within the COSUS project was to specify product-related factors that are responsible for the generation of food waste, in both the store and the home situation.

#### 2.1.1. Product-Related Factors: Experimental Approach

Product cues and properties that lead to a non-selection of individual foods in supermarkets or to discarding foods at home were investigated by complementary qualitative and quantitative approaches. In a first stage, 83 consumers from the five COSUS countries participated in a total of 10 focus group sessions (two sessions per country), with the aim to identify facilitators and barriers for the selection and consumption of visually suboptimal food. Each focus group session comprised four successive exercises: (1) *Projective mapping*: individual projective mapping exercises of apple and carrot images of varying shape and freshness, followed by group discussion of the resulting maps; (2) *Home photos*: presentation and discussion of photos of foods that the participants knew they will discard from their kitchen; (3) *Faulty products at home and in the grocery store*: discussion about seven products presented in a “standard” and a “suboptimal” version (chocolate, bread, potatoes, carrots, pickled cucumbers, muffins, yogurts and milk); (4) *Brainstorming of ideas for store interventions*: idea generation and discussion, first as a grocery store manager, then as a consumer. A common structured interview guide was developed and utilized by experienced moderators in all countries. Sample products in the faulty products discussion were selected and manipulated in a standardized way from each local market, with the exception of the chocolate (fat bloom defect) and muffins (over-baked defect), which were provided by partner Technische Universität, Dresden, for all countries.

In a second stage, 4214 consumers from the five COSUS countries (for socio-demographics, see Table 1) participated in an online choice experiment [8]. Consumers had to imagine either that they were in a supermarket, ready to buy products (the supermarket condition), or that they were in their home, ready to consume (the home condition). They then saw six pairs of foods, each consisting of a picture of a visually suboptimal food (e.g., an apple with a spot, a bent cucumber, milk or yoghurt close to or passed the best before date, a dented carton of juice or broken biscuits) and a picture of its optimal counterpart. For each pair, the consumers indicated which one they would choose to buy (given an identical price, in the Supermarket condition), or which one they would choose to consume (in the Home condition). They also indicated what the lowest acceptable discount would be for them to purchase the suboptimal product (in the supermarket condition) or how likely it was that the suboptimal product would be discarded (home condition).

#### 2.1.2. Product-Related Factors: Major Findings

The qualitative sessions revealed insights at several levels; we will here focus reporting on three selected aspects, namely: consumer perception of food waste; the motivators for discarding foods; and applied strategies to avoid food waste. Firstly, we observed a wide diversity in the images of foods that was expected to be wasted from the participant kitchens both in terms of food categories (bakery products, dairy products, fruits and vegetables, meat products, condiments, spices and sauces) but also in terms of shelf life: the products ranged from rotten vegetables to full quality articles such as canned foods or dried spices. It was explained by our participants that the latter type of items could remain in cupboards for months or even years until one acknowledges that these will never be consumed due to a lack of envy and/or a lack of routine on using this type of items. Such imperishable-yet-discardable foods had typically either been acquired during supermarket promotions, or had been offered during visiting friends or family, that is to say that these items did not belong to the usual food repertoire of the respective consumer. It is also interesting to note that, for some participants, throwing rotten vegetables or mugged sour cream away would not be defined as wasting food, since “one cannot eat what is rotten”. This type of thinking may explain underestimated self-reports of food waste observed in the literature. Furthermore, while the need for discarding clearly deteriorated food was unanimously understood, individual variations emerged on the need for discarding food that only had lost its freshness. On the one hand, some of the participants would be too worried with respect to food safety and “not take the chance” to eat a product that has been lying a little too long in the refrigerator. On the other hand, several of the participants had clear strategies and routine recipes to make use of, for example, dry bread, brown bananas, milk past the best-before date or forgotten cooked ham in an open pack. These strategies often involved heat treatment, allowed neither to compromise on food safety nor on palatability, and were often anchored in family habits passed on from the former generation. Contrary to our expectations, a few participants seemed to be strictly concerned about date stamps especially on refrigerated products at home, and another reported concern was that the date on the package is a poor guide once the meat pack or the dressing container had been unsealed. Look—smell—taste strategies to evaluate such products were not always habitual among participants who felt unable to evaluate food safety by their senses. Finally, the focus groups highlighted a clear distinction in food waste-related behavior at home and in supermarkets, as participants were much more relying on date stamps and beauty flaws of products in the supermarket-setting discussion (i.e., while judging what may become theirs) versus in the home-setting discussion (i.e., while judging products that they supposedly owned already). We decided to further investigate this distinction as a part of our following quantitative study.

**Figure 2:**
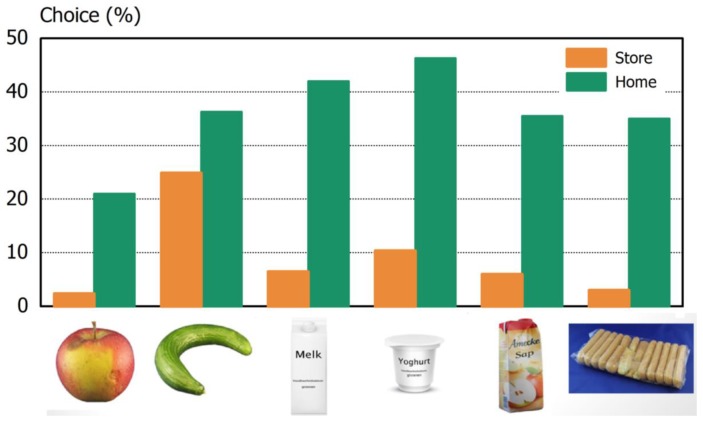
Choice of the suboptimal food differs between the supermarket- and the home-setting (*n* = 4214). Each pair of bars is associated with the respective product images below.

The findings of the cross-national choice experiment [8] converged with the findings from the qualitative research. There were some variations in consumer selections of suboptimal foods which depended on the type of suboptimality (appearance deviation, date labeling deviation, or a packaging deviation), and on the setting the consumers found themselves in. For each suboptimal product, consumers selected it less frequently when being in a supermarket compared to being at home. Most notably, only the buckled cucumber with its appearance deviation was selected by more than 10% of the consumers in the supermarket setting (namely, by 25% of the consumers); in the home setting, suboptimal products were selected by between 21% and 47% of the respondents (Figure 2). For all products, it appeared that associations with the product as being (un)attractive and/or as being (un)safe to consume played a distinct role in the decisions to buy or consume the suboptimal product. Consumers needed a higher discount to purchase products that were perceived as unattractive or unsafe, with indicated discounts ranging from 24% for the cucumber with its appearance deviation to 67% for an apple with a brown spot. Moreover, the likelihood of suboptimal products ending up in the bin increased with higher unattractiveness and unsafe associations, ranging from a 14% chance for the buckled cucumber to 36% for the brown-spotted apple. It therefore seems relevant for both research on foods and for supply chain actors to distinguish between appearance deviations in terms of shape, color, and size.

In the choice experiment, suboptimal product choices appeared to depend on a number of consumer characteristics, and the influence of these consumer characteristics in turn depended on the setting. In the supermarket setting, consumers were more likely to purchase suboptimal products when they had higher perceived consumer effectiveness, and when they found the food waste issue relatively more important. In the home setting, consumers were more likely to consume suboptimal products when they were more committed to environmental sustainability, and more engaged in grocery shopping and cooking. This finding converges with the idea that consumers more experienced with food might have more strategies and routine recipes to make use of suboptimal products. Surprisingly consumers were, in both settings, more likely to choose suboptimal products when they were younger; this seems to contradict existing food waste research that showed that younger consumers waste more food. It might therefore be possible that findings on consumer food waste behavior do not automatically translate to consumer behavior towards suboptimal products.

### 2.2. Person-Related Factors

Attitudes towards suboptimal food might be linked to emotions, the motivation to be sustainable, education, and other internal and external factors such as information; consumer segments might differ in their overall food-related lifestyle [9]. For instance, while some consumers may accept spots on organic apples, they may not do this on conventionally grown apples [10]. Therefore, consumer attitudes towards suboptimal food and motivations for accepting or discarding suboptimal foods should be analyzed along with general attitudes towards sustainable food consumption. The analysis of consumer psychographics and behaviors related to food waste avoidance and acceptance of suboptimal foods may therefore lead to the identification of consumer segments.

Consumers may indicate that they will accept and eat certain foods, but this is not the same as actually buying these products once they are in the store [11]. Acceptance may be high when the product is free or cheap, particularly for low income groups [10], but the consumer may not be very willing to pay for sustainable foods, in particular without any additional environmental encouragements [12,13,14]. Consumers with the same sociodemographic characteristics might be expected to be wasting food and discarding suboptimal food due to the same reasons. However, in the complex issue of consumer-related food waste [5,6], the psychographic characteristics play an important role, as well as does the interplay between sociodemographic and the psychographic, attitudinal consumer characteristics. In other words, seemingly similar consumers might waste food because of completely different reasons. Therefore, we tested the willingness of consumers to pay for sustainable foods in response to information and in relation to their personal attitudes and motivations, attitudes towards and trust in the retailer and branding. In addition, current strategies that motivate consumers to pay for sustainable food in supermarkets were evaluated by analyzing actual sales data. A consumer segmentation study was conducted to identify consumer groups of difference and similarity across the various countries, and to develop policy recommendations which can be expected to match and target each group more efficiently.

#### 2.2.1. Person-Related Factors: Experimental Approach

In the same cross-national survey as referred to above, consumers were also asked to assess a range of 54 statements describing their ‘food-related lifestyle’, including statements related to food waste. The food-related lifestyle measure represents consumer activities, interests and opinions in the domain of food in everyday life, and covers the following areas: (1) *Purchasing motives*; (2) *Quality aspects*; (3) *Consumption situations*; (4) *Ways of shopping*; and (5) *Cooking methods*. The measure was based on the internationally tested food-related lifestyle scales [9,15] which have been repeatedly used in different cultural contexts (e.g., [16]) and topics (e.g., [17]). With the help of the expert interview and literature study [5] and focus group sessions conducted as part of the project, the measure was adapted to the topic of food waste. Through exploratory factor analysis, the cross-country factorial structure of the data gathered from the online survey was determined [18], and a two-step cluster analysis process [19] was applied to identify the consumer segments. These were then characterized on knowledge of the extent of food waste, relative importance of the food waste issue, self-reported estimation of own food waste in five food categories, frequency of choosing the ‘optimal’ product across six categories, and sociodemographic characteristics, using ANOVAs for multiple group comparisons, and testing for between-group differences with post-hoc tests.

#### 2.2.2. Person-Related Factors: Major Findings

Based on 31 statements, found to have a common and similar meaning across the five countries, five segments of consumers that are distinct in their food (waste)-related lifestyle patterns emerged through the analysis. Various dimensions that describe food involvement (that is, an interest in food such as enjoying cooking, deriving self-fulfillment from the task, or seeking quality food such as organic or healthy food) were of importance, as were dimensions on planning, meals as social events, and price orientation. The findings show that differences in self-reported food waste and food waste-related behaviors can be uncovered, and suggest that there is a relationship between food-related lifestyle patterns and food waste (see [20] for more details). The first segment called the *Involved socializers* was characterized by high food involvement and a frequent usage of meals as a social event, while the second called the *Un-involved* was characterized by the opposite, a low food involvement across many dimensions (Table 2). The *Price-oriented* (segment #3) particularly focused on price during shopping, while the *Price-dismissive* (segment #5) gave a relatively low importance to price, and showed a medium involvement with food. The *Well-planning* (segment #4) were found to be nearly as involved as the *Involved socializers* but, in addition, specifically characterized by planning meals several days ahead. The first and the second segment reported a greater amount of food waste at home than the others, but the first considered food waste to be a more important issue than the second segment. Of all segments, only segment #4 was significantly more likely to use suboptimal food first while at home. As policy recommendations for tackling the food waste issue, it becomes apparent that the *Un-involved* are not motivated in the topic, thus strategies like for example nudging towards food waste avoidance are needed here [21]. Consumers who have greater social interaction related to meals and who are very involved (segment #1) can be engaged as multipliers, or take part in public events or socially interactive campaigns. Price-oriented consumers, in turn, can be expected to react positively to price incentives such as price-reduction of suboptimal food [22] and food redistribution into alternative retail [23]. Price-dismissive consumers will more likely be open to invest money to avoid food wastage, such as spending more in a restaurant or supermarket that engages in own action to reduce food waste in the supply chain [24]. Consumers such as those from segment #4 might waste relatively less and have the needed food planning handling capabilities [25], thus, involving these consumers to share their experiences and competences would support the proliferation of strategies to reduce food waste in consumer households.

## 3. Communicating Food Waste Reduction to the Consumer

### 3.1. Product Cues Attracting Attention on Suboptimal Foods

Producers and retailers provide the consumer with information about a particular food. The name of the product, nutritional information, any claims, and the best-before date are examples of information given on food packages. Specific messages related to sustainability such as regional origin or organic food labels may encourage consumers to make a more sustainable choice (e.g., [12,26]). Such information is however not noticed by all consumers, and not all types of information are effective in changing consumer behavior [27,28]. One task in the project was to test the attention paid to information and the influence of the type of information (direct versus subtle), presentation of information (e.g., on the package, in store, through other media), and the content of the message on consumer acceptance of suboptimal foods.

#### 3.1.1. Attracting Attention: Experimental Approach

In an eye tracking study [29], 30 subjects (21 female, 40 ± 14.6 years) visually inspected suboptimal foods with or without a specific message among impeccable ones in a purchase or discard decision task while their eye movements were recorded. Pictures of foods were arranged in a 3 × 3 grid, with the central position left empty to avoid a central gazing bias [30,31]. Thirty-two pictures served as filler items, whereas 8 test foods (cucumber, banana, piece of butter, juice carton, carrot, apple, milk carton, pile of cookies) were available in 6 variations: impeccable; in suboptimal appearance (e.g., a buckled cucumber, a pile of partially crumbled cookies, an apple with a small brown spot); in suboptimal appearance with either of two labels with varying message (“Small in price” or “Great in taste”); and with the respective message labels in different color (green vs. red).

**Figure 3:**
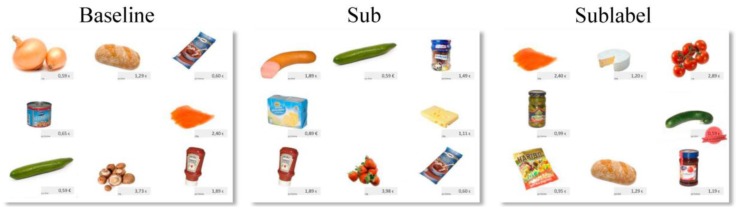
Examples of matrices for the baseline, sub (a damaged package of butter), and sublabel condition (a buckled cucumber with a red price reduction label). The figure is part of Figure 2 (amended version), published in Helmert et al. [29], Have an eye on the buckled cucumber: An eye tracking study on visually suboptimal foods. Food Quality and Preference 60 (2017) 40–47, with permission from Elsevier. The study was conducted in Germany so a comma is used as decimal indicator.

Three different test conditions were deployed: *Baseline grids*, each containing one of the eight impeccable test products (cucumber, banana, piece of butter, juice carton, carrot, apple, milk carton, pile of cookies); *Suboptimal grids*, with the test products in visual suboptimal appearance; and *Sublabel grids*, containing a suboptimal test product with either message on either color label (for an example, see Figure 3).

The subjects were informed that, after inspecting each of 136 grids that showed 7 filler items and one test item in one of its six modifications, they had to answer one of two possible questions: “Which of the objects would you like to keep in your shopping cart?” (Purchase option), or “Which of the objects would you discard from your shopping cart?” (Discard option). After the subjects signaled that they are ready, the grid was removed, and one of the questions was randomly presented. A single trial was finished when one of the eight positions of the matrix was selected as response. The order of the matrices was randomized for each participant. Dependent variables were time to first fixation, total fixation duration, and food choice.

#### 3.1.2. Attracting Attention: Major Findings

The results show that the design changes indeed attracted attention towards suboptimal foods. Time to first fixation and total fixation duration were analyzed separately by repeated measures ANOVAs with condition (baseline, suboptimal, sublabel) and item (filler, relevant) as independent variables. We found significant interactions of item and condition. Depending on their condition, eye movements falling on relevant items differ from those on the filler items. Therefore, in a second step, differences in both measures between relevant and filler items were calculated with condition (baseline, suboptimal, sublabel) as independent variable. Significant differences were obtained for condition with the shortest time to first fixation for the sublabel condition, intermediate for the suboptimal condition, and longest time for the baseline condition. The analysis of total fixation duration also yielded significant differences between the conditions with an increase from baseline via suboptimal to sublabel. Within the sublabel condition, only color yielded differences between the design variations, with red resulting in longer total fixation durations. No significant influences were found for the time to first fixation. The content of the message was, however, irrelevant.

Additionally, we inspected the proportion of test stimuli that were chosen. Question type (discard vs. purchase) was added as independent variable. For the discard question, there was no significant difference between the conditions. For the purchase question, condition differed significantly. Purchase decisions declined for the suboptimal as compared to the impeccable items in the baseline condition. However, when presented with differently designed price badges, a positive trend to purchase the suboptimal items was obtained.

**Figure 4:**
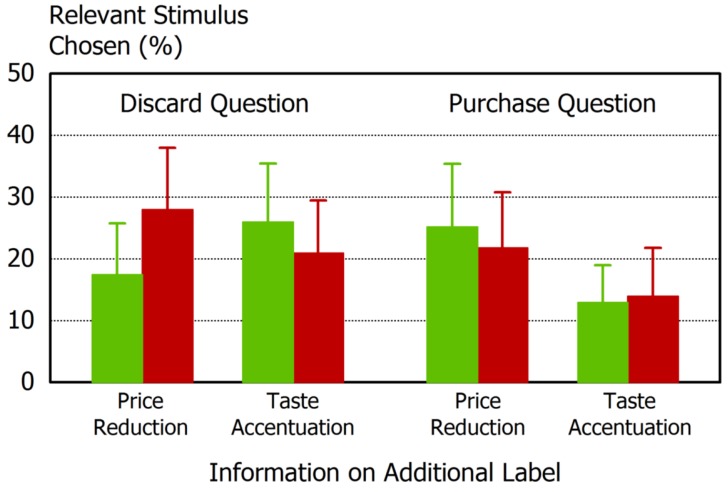
Interaction of label color (green, red) and label information (price, taste) in trials with the discard question, and in trials with the purchase question; error bars represent 95% confidence intervals. Redrawn after Helmert et al. [29], Have an eye on the buckled cucumber: An eye tracking study on visually suboptimal foods. Food Quality and Preference 60 (2017) 40–47, with permission from Elsevier.

We also analyzed effects of information and color within the sublabel condition (Figure 4). For the discard question, neither color nor information reached the significance level; the information/color interaction was however significant. Products with red price information labels were most often chosen, followed by green/taste combination. Red/taste and green/price received lowest choice probabilities. For the purchase question, only a significant influence of information was observed. With reduced prices, purchase decision was substantially higher than with the taste information.

Our results emphasize the importance of highlighting suboptimal foods to attract consumer attention and force respective purchase decisions. Therefore, supplying visually suboptimal foods in stores should be embedded into efforts to attract attention towards these products, as selling visually suboptimal food might positively impact waste balance along the food supply chain.

### 3.2. Expectation and Perception of Suboptimal Foods

Wastage of suboptimal foods may also be caused by low expectations with respect to sensory quality. As they cannot use their oral system in purchase situations, consumers rely on product appearance and extrinsic information when making their decisions. Suboptimal sensory appearance (e.g., odd shape, spots on fruits or vegetables) is perceived by vision, is processed affectively, and has been shown to strongly drive consumer responses [32]. Likewise, an imminent best-before date on the package has been shown to signal reduced freshness and may hence affect sensory expectation. Tsiros and Heilmann [13] stated that consumers use the best-before date as a way to judge food safety, and that they pay more attention to such a date when the product is ‘risky’, e.g., in the case of meat or fish. In many cases consumers do not distinguish between the best-before and the use-by date, the latter being an absolute for food safety, while the best-before date is some sort of guarantee given by the producer that the food has still its typical characteristics [28].

Numerous studies demonstrated that sensory expectations are a key factor in food choice, and that poor expectations mitigate the hedonic experience of otherwise much enjoyed products (e.g., [33]). As the food store (or the refrigerator of the consumer) usually offers nicer and fresher alternatives, suboptimal foods remain on the shelves (or in the fridge). It is, therefore, important to investigate the role of sensory expectations on the acceptance of suboptimal food.

#### 3.2.1. Expectation and Perception: Experimental Approach

In a first experiment [34], we systematically manipulated environmental conditions during Cavendish banana storage to obtain regular ripened bananas (ripeness degree, RD 5) and more ripened, visually suboptimal bananas (RD 7). A forced-choice paired comparison test with 35 trained participants was initially conducted to identify sensory properties out of a pool of twelve flavor, taste, and texture attributes that are significantly different. A total of 233 participants (140 male, 22.2 ± 3.1 years) took part in the main study and were randomly distributed to four experimental groups. Some of the participants received a peeled banana of either ripeness degree to rate perception only (group 1, group 2): They evaluated overall liking using a 9-point hedonic scale, the intensity of the sensory attributes using five-point just-about-right (JAR) scales, and purchase intention using a seven-point scale. Other subjects received an unpeeled banana of either ripeness degree to rate expectation and, after self-peeling, also perception (group 3, group 4). They started with the rating of expected overall liking, expected intensity of sensory attributes, and purchase intention. After that, they were asked to peel and taste the banana and to perform the respective ratings again.

In a second study, we used strawberry yogurt that differed in the best-before date by two weeks. A preliminary triangle test with 36 participants was conducted to identify differences between the yogurts (fresh, two weeks; old, one day apart from best-before date). In a Check-all-that-apply test, 58 subjects identified the attributes to be used in sensory analysis. The 290 participants of the main study (120 male, 32.7 ± 16 years) were assigned to five groups: two groups received either ‘fresh’ or ‘old’ yogurt without knowing the best-before date; they tasted and rated overall liking, purchase intention, and sensory attributes using JAR scales (perception only); two groups who had prior knowledge of the best-before date (‘fresh’ or ‘old’) rated similar, both before (expectation) and after tasting (perception); and participants in a further group were additionally primed by receiving a brochure containing ‘golden rules’ to reduce food waste.

In a third study, a preference test with chocolate was conducted. 61 participants received two pieces of chocolate—one with and one without a fat bloom. Fat bloom was manipulated by exposing the chocolate to elevated temperature, so that a whitish coating appeared on the surface [35]. Participants had to evaluate before tasting which piece of chocolate they think will taste better. After that, they tasted the chocolate and were asked which piece of chocolate tasted better.

#### 3.2.2. Expectation and Perception: Major Findings

One element that is relevant when making the choice whether to accept or discard suboptimal foods is current sensory expectation and previous experience with the respective product. It was possible to demonstrate that a positive experience with suboptimal foods positively affects consumer intent to purchase and consume suboptimal foods.

The paired comparison test of the banana study revealed five attributes that participants rated more intensive for a particular ripeness degree than for the other one. These attributes were grassy flavor, banana flavor, sweetness, firmness, and mealiness; these attributes were further used in the just-about-right evaluation. In the main study, expected overall liking and purchase intention were significantly lower for the overripe bananas. Purchase intention was still significantly different after tasting the different bananas, whereas no difference in overall liking was observed. Concerning the sensory attributes, penalty analysis revealed that only the firmness of the overripe bananas was still not just-about-right after tasting. For suboptimal bananas, the results demonstrate a positive relationship between sensory perception, overall liking and purchase intention.

**Figure 5:**
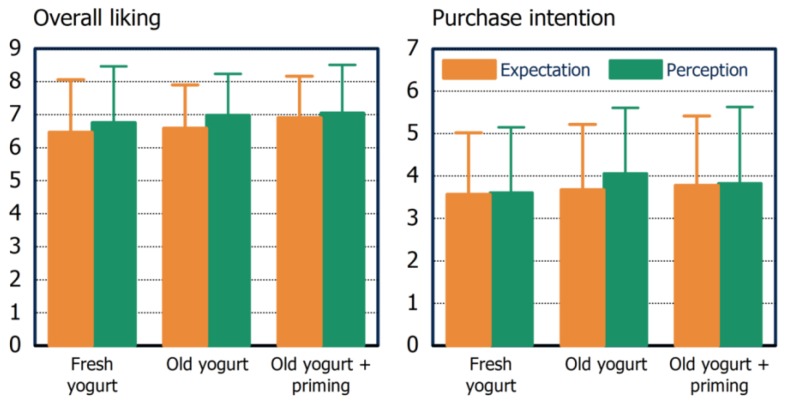
Means of overall liking and purchase intention before (expectation; best-before date was known by the participants) and after tasting yogurt (perception).

In the yogurt study, the participating subjects were not able to identify the deviating sample in the initial triangle test. Thus, any differences in the main study can be attributed to the best-before date as such and not to any between-yogurt differences. In the Check-all-that-apply test color intensity, thickness, creaminess, flavor intensity, sweetness, and sourness were identified as relevant sensory attributes. The main study showed a higher overall liking and purchase intention after tasting compared to the before-tasting ratings in the groups that were aware of the best-before date. This was even true for the group that received ‘old’ yogurt, meaning that lower expectations towards this product disappeared after tasting (Figure 5). In other words, a positive experience with the ‘old’ yogurt increased both measures. However, no additional priming effect was observed. JAR analysis revealed a too-high sweetness and too-low creaminess which had a negative influence on overall liking in all experimental groups. However, in the priming group, the negative impression of creaminess did not influence overall liking meaning that this property should be stable over storage in the household. A clever choice of priming strategies may help to increase consumer acceptance.

The results of the chocolate study showed that 59% of the participants expect the chocolate without fat bloom as being tastier. Even after tasting more than half of the participants (54%) perceived the chocolate without fat bloom as being tastier—although there was no objective difference between the chocolates (in terms of the date of purchase and time of storage). 

The findings of all three studies indicate that tasting experiences with visually suboptimal foods positively influence overall liking, purchase intention, the perception of sensory attributes, and preference. Convincing consumers that those foods are still tasty is of high relevance for recommending different ways of communication.

### 3.3. Messages and Channels to Communicate Suboptimal Foods

The knowledge and insights generated in the analytical part of the project was used to develop and test interventions aiming at motivating consumers to purchase and accept suboptimal foods in stores and in their households. For doing so, three approaches were used: different information material that was provided on household level by different information channels; a mobile phone app that was tested by selected consumers; and an in-store intervention where different messages were tested to identify the potential effects on consumer behavior.

#### 3.3.1. Communication Channels: Experimental Approach

**Figure A1:**
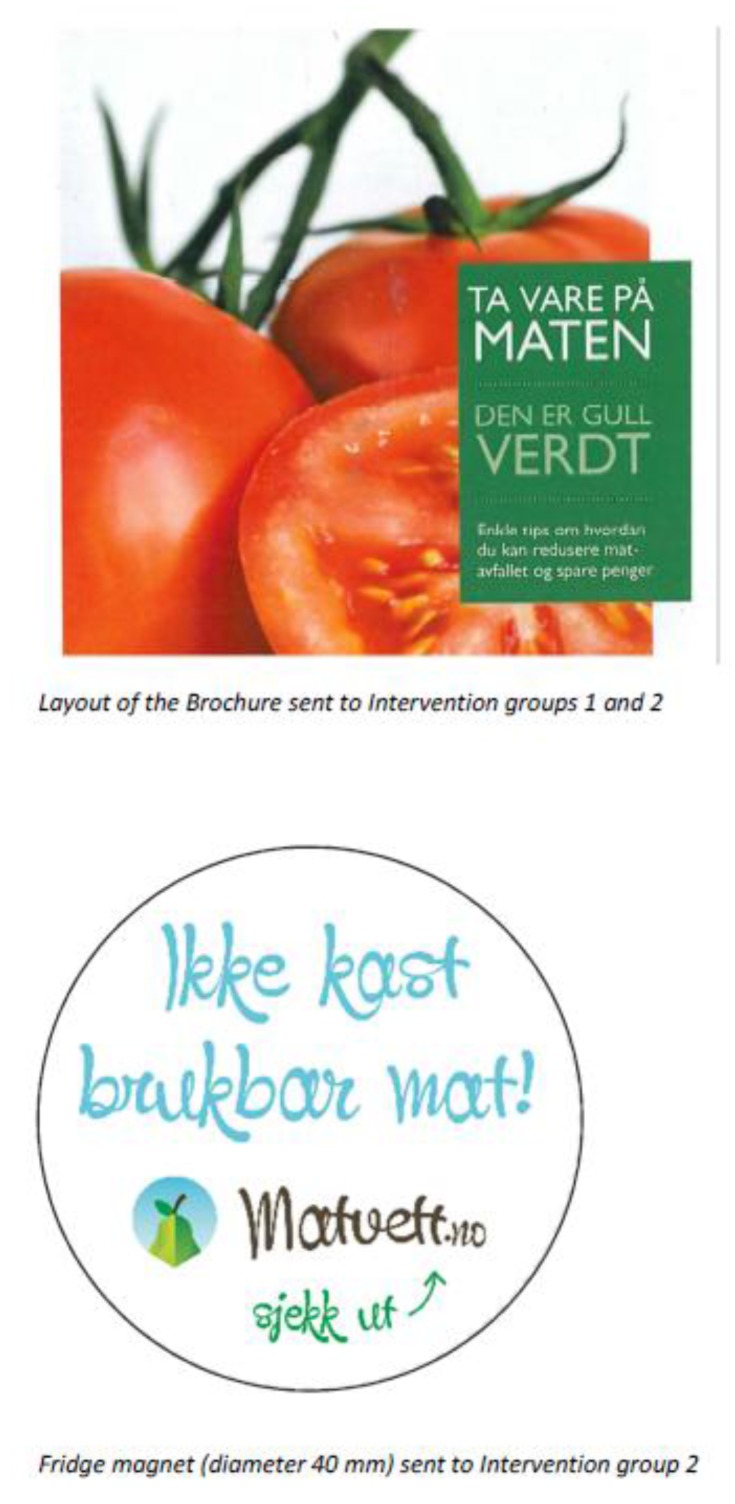
Material used in the Household Intervention.

One of our intervention experiments aimed at investigating the effects of several communication channels against food waste: a brochure, a fridge magnet, a website and a Facebook group on self-reported suboptimal food choices and behaviors. The study was conducted in Norway with a total of 622 respondents, age between 25 and 50 years. The respondents were split into three groups: Intervention group 1 (*n* = 207) received a brochure by post which, among other, provided tips on how to avoid food waste; Intervention group 2 (*n* = 205) received the same brochure and a fridge magnet with the message “Don’t waste usable food!” intended as daily reminder for priming against food waste (for some information on this material, see Figure A1). Group 3 (*n* = 210) served as control and did not receive any material prior to filling a web questionnaire. The brochure and the magnet invited to visit the website, while the website invited to join the Facebook group, this being the official website and Facebook pages of a major national non-profit organization against food waste (www.matvett.no). Three weeks after having sent the material to groups 1 and 2, all groups answered a web questionnaire including a choice task on optimal versus suboptimal foods (the same task as in the Cross-national survey) conducted in an evoked home-context condition, reported behavior within the last 2 weeks corresponding to the 10 practical recommendations included in the brochure, selected food-waste related lifestyle items (selection based on results from the cross-national survey), questions regarding visits to the website and adherence to the Facebook group, and socio-demographic parameters.

**Figure A2:**
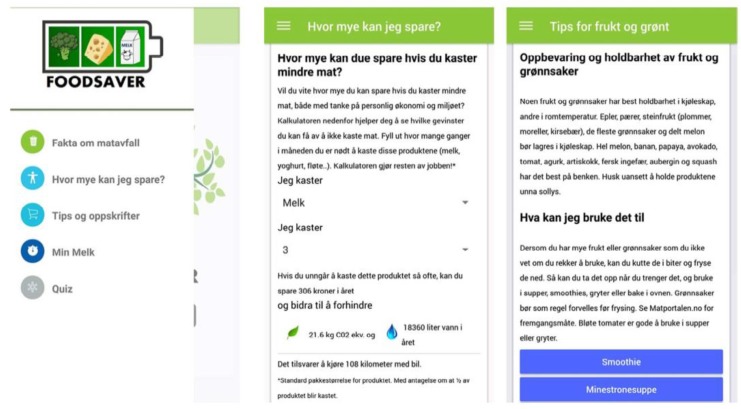
Screenshot of the app used in the Intervention.

For the final household intervention, a mobile phone app was created that provided several communication messages to consumers at home. All communication focused on increasing acceptance of dairy products at or past the best-before date, as consumers already show some acceptance of such products. The communication messages aimed to help consumers to overcome any uncertainties they have about food safety, about how to cope with dairy products past the best-before date, and to motivate them to taste the foods before eventually discarding them. The app also included messages about how not wasting food contributes to environmental saving, as well as helps to save money spent for food (Figure A2). The content of the app was fine-tuned using results of five group interviews with 13 participants. Three groups of participants (*n* = 50 in each group) were asked to evaluate an app over a period of 2.5 weeks. Before the participants started the evaluation, they were asked to answer questions about their food-related life style including attitudes towards suboptimal foods, the use of mobile phones and technology, and sociodemographic questions. This questionnaire allowed for balancing the three groups with respect to gender, age, education, number of children in the household, and frequency of mobile phone use, and provided a baseline regarding attitudes towards suboptimal foods. To test how the app features influence attitudes, the three groups received different versions: (1) the full app that was specifically focusing on dairy products; (2) a smaller app version that was more generally about food waste reduction; (3) the full app, but with screenshots printed on paper rather than an interactive version. The latter group was included as not much research has been done on the effectiveness of apps as tools for communication. The final evaluation consisted of a questionnaire including questions about content and design of the app, perceived usefulness of the functions, and also including some questions that allowed a comparison of attitudes to suboptimal foods before and after use of the app.

The objective of the final store intervention was to show whether and to what extent communication messages the potential to encourage a sustainable food choice have. Based on the results of previous work, three different messages were used: One message that refers to taste (“I might not have the looks—but I am super tasty! Convince yourself!”), one message linked to sustainability (“Grab me! Too good to waste. For the sake of the environment!”), and one message was about the price (“Get me for a special price! Save money!”) and referred to a 50% discount. The fourth test condition served as control (no message presented). Bananas were chosen as material as these are part of the usual product assortment of the canteens in which the experiments were carried out (a cafeteria of the university, a cafeteria of a private research institute, and a cafeteria in the library). On study days, only suboptimal bananas with brown spots were sold. The messages were positioned close to the offered samples. Each message was tested in each location at lunch time (10:00 a.m.–3:00 p.m.) in a randomized manner. The study consisted of two parts: First, the researcher observed the cash area and waited until a consumer chose a banana. Second, after having paid, the respective buyers were asked to fill in a short questionnaire. In sum, 229 participants filled in the questionnaire after having bought a banana.

#### 3.3.2. Communication Channels: Major Findings

The communication channels study revealed that brochures and fridge magnets did not have any detectable effect on consumer attitudes, self-reported behavior and suboptimal food choice, with an average of 2.7 suboptimal foods chosen out of 6 across all three groups. It is likely that effects would have been observed if the survey had been conducted shortly after sending this material to the respondents. However, we wanted to know if such material could trigger lasting effects, after three weeks time. Furthermore, respondents were significantly more likely to visit the website if they had received the brochure, and even more so if they also had received the fridge magnet (19% of respondents in group 1, 23% of respondents in group 2 and 9% of respondents in the control group visited the website). Similarly, significant but small effects were also observed on new Facebook followers (5%, 4% and 2%, respectively). Finally, respondents who had received the magnet in addition to the brochure stated that, with respect to food waste reduction, the brochure had influenced their own and the actions of other members in the household to a higher degree. Respondents who visited the website and/or followed the Facebook group reported higher effectiveness of the brochure and the magnet on their actions than respondents who did not use these channels. Conclusively, communication channels such as brochures and magnets cannot be considered as convincing tools to impact consumer attitudes, food waste reduction behavior and suboptimal food choices in the targeted 25–50 year old age range. We may note, however, that these tools may work as an entry into increasing consumer awareness and interest for other channels such as websites and Facebook initiatives. This could in turn slowly impact consumer food waste reduction behavior.

Due to technical difficulties, the mobile phone app was evaluated rather negatively. However, some interesting results were obtained: the intervention (regardless of group) had a small positive effect on attitudes towards suboptimal foods and on self-assessed food waste behavior during the intervention period, suggesting that the messages of the app reached the participants. There was a large variation between individuals in perceived usefulness of the app (independent of technical difficulties, and backed up by results from the group interviews): some individuals indicated it as being low, as they already have good knowledge about how to avoid waste and what to do with dairy products past the best before date, while other participants indicated that such an app is very useful. This is likely related to characteristics that separated the consumer segments earlier in the project: those with high food involvement/planning are less likely to need such an app than consumers that lack planning skills or knowledge, but have a moderate food involvement. Although the app in itself did not majorly impact the acceptance of suboptimal foods, the results do suggest that apps may help some segments of consumers to be more aware of food waste and to gain knowledge about how to use suboptimal foods in the household.

By testing messages (taste, sustainability, price, no message) in a real consumption context, the store intervention revealed a clear influence of the messages on consumer behavior. For each condition/message between 2500 and 3000 consumers passed the cashier. A total of 96 consumers (3.5%) bought a banana when the sustainability message stood next to them. Thus, this message was the most efficient one. A lower price was only for 80 consumers (2.7%) an incentive to buy the banana, and only a few bananas (19, 0.7%) were sold when the taste message was presented. In the next step we were interested in whether the participants recognized a message and whether they remember the right message. On average, 46% of the participants recognized the right message with the sustainability message having the highest recognition value compared to the other messages (68%).

### 3.4. Price-Reduction to Sell Suboptimal Food

The expert and literature overview indicated that price-related measures were regarded as one possible and potentially powerful measure to reduce the waste of suboptimal foods: Price is given ‘part of the blame’ for food waste, e.g., when the prices of foods are too low to ensure valuation of the food, or price-related marketing tactics trigger over-purchase [5,36]. However, this also entails the acknowledgment of price as a powerful tool. In addition, research also indicates that price orientation of consumers not necessarily leads to a larger amount of waste (e.g., [37]). As a measure of steering consumer perception and choice, the reduction of price increases the incentive to choose the food relative to normal priced foods, and it improves the price-value relation of the foods. Given this, price-related actions are regarded as one possible direction for reducing food waste [38].

To explore the effectiveness of reducing the price of suboptimal foods (otherwise wasted in the store) to ensure that it is sold, a number of approaches were used. The research took place in Denmark as a case study, given this emerged to become widespread during the course of the project. Firstly, a mapping study was conducted taking stock of the breadth of approaches and actions across different food categories and retailers in Denmark, using store-checks and mystery shopper interviews of store-personnel, and a content analysis of the Corporate social responsibility reports of the retailers and the website descriptions of their actions. Then, a qualitative consumer study using accompanied in-store shopping interviews was set-up with the aim to identify the considerations of consumers in-store and during purchase when they are faced with suboptimal foods that are reduced in price and that bear respective stickers that communicate food waste avoidance and price reduction. Thirdly, a quantitative consumer study using online experimental work researched consumer reactions to sticker communication messages in the context of different retailers, to explore the communication which is most appealing, and the role of familiarity with the approach. 

During the mapping study of retail actions in Denmark, twenty-two food waste avoidance actions were identified, categorized as (1) Price-related or pricing; (2) Product-related; (3) Unit-related; (4) Communication about food waste issue; (5) Collaboration with other actors; and (6) In-store management [39]. It emerged that nearly all Danish retailers applied best-before date based pricing [40] in at least some categories, but that the approach in communicating the action, placing the items, and the layout of the stickers varied widely. Supermarkets also varied in the way they exploited price promotions, with one retailer having abolished multi-item offers to avoid food waste [38], while others used it a lot, despite their food waste avoidance actions elsewhere. In general, store managers assessed that selling price-reduced suboptimal foods is efficient—with 9 out of 10 items sold—and the measure appeared to have become a standard approach, quite often communicated in the context of food waste avoidance.

Through the qualitative interviews with 16 consumers, it was explored which kind of factors consumers consider, discuss and take into account during shopping when confronted with price-reduced suboptimal foods. The results showed that consumers appear to carefully assess their ability to consume the price-reduced suboptimal foods at home, even though it has a shorter time frame unto best-before date or is suboptimal in some other way. As factors, consumers mentioned product-related factors of package unit, date issues, and product quality. They saw this in interaction with household-related factors of their freezing and storage possibilities, the demand and size of the household, and whether it was possible to integrate the food in a meal. Even though food waste avoidance was evident in the consumer interviews, the main reason for choosing price-reduced suboptimal food was their actual possibility to use the item. Reluctance towards any waste of food—perceived majorly as a waste of money rather than a waste of environmental resources—was observed among consumers [22]. 

The quantitative experimental survey among 842 Danish consumers focused on foods nearing the best-before date [41]. Giving it was designed to mimic the current market context, the findings can be expected to be rather externally valid and applicable. Experimental situations shown to the respondents differed in which retailer they were presented to ‘shop’ at, whether the sticker was communicating the price reduction of the food waste avoidance, and the type of product (in terms of category, and the product being organic or not). The results indicate that the communication has a minor impact on choice when compared to the familiarity with the context (whether it is a retailer and a sticker the consumer in question is familiar with). In particular, the perceived quality of the item and whether or not the consumer expects to be able to consume it all at home, explained likely choice. Thus, the findings imply that the layout of the sticker and the content of the communication and motive used is of much less importance. For retailers and policy makers wanting to ensure that consumers adopt the approach of price-reduced suboptimal foods on a broad scale, it should be a priority to ensure that it is broadly applied so that all consumers are familiar with it. In addition, to increase choice likelihood, it is of special importance to ensure suboptimal foods quality, and to provide information and tips on how to consume it.

## 4. Ways of Reducing Food Waste along the Food Supply Chain

Strategies that encourage consumers to accept, buy and eat suboptimal foods can only be successful when they are implemented by different actors of the food supply chain. Retailers and food producers play an important role in implementing such strategies. In addition, these actors are responsible for part of the suboptimal food waste. Perishable goods make up almost 70% of the total products in grocery stores, and in-store spoilage is around 4.5% due to not being sold on time or due to imperfections [13]. Total food waste in the agri-food supply chain is around 35% [42]. Strategies to encourage sustainable consumer behavior that are initiated by food supply chain actors can only be successful when consumers accept the strategies, and when retailers and food producers can implement such strategies without being limited by regulations, technical limitations, or financial or marketing reasons. Therefore, we also identified the barriers and opportunities for improved handling of suboptimal food products in the food supply chain.

### 4.1. Business Potential and Barriers of Suboptimal Foods

To take stock of the drivers of successful actions of market actors and stakeholders, the research team conducted a case study [43] across European countries and different types of initiatives. The study chose 26 initiatives which aim to reduce consumer-related food waste, and explored these by data triangulation with expert interviews, content analysis of published material, and reports or media articles. In addition, the perspective of key success factors, understood as factors related to (a) Macro-environment (e.g., political, economic, environmental, social, technological, or legal); (b) Micro-environment (e.g., supply chain partners, customers, competitors); or (c) Internal factors (e.g., strategy, competences, resources) of the organization, and factors key and crucial to the success [44,45], was employed.

Three different types of food waste avoidance initiatives were identified [23]: *Information and capacity building*, *Redistribution*, and *Retail and supply chain alteration*. An entrepreneurial spirit or internal motivation of a main actor in an initiative was observed in a number of the initiatives. As success factors of particular importance, the following factors emerged: Collaboration between stakeholders, timing and sequence of initiatives, competencies that the initiative is built on, and a large scale of operations. The findings allow recommending that future initiatives should take example of previous cases in the respective type of action, in particular trying to consider the right partners to collaborate from the start, time the initiative in good synergy with other macro-environmental trends, and aim to achieve a high scale of operations in rather short time.

As food waste (reduction) behaviors might be different from behaviors towards suboptimal foods, we also conducted thirty-three interviews with producers, producer organizations, and retailers to study their behaviors towards suboptimal foods. Multiple scholars have mentioned that the existence of (European Union) specifications concerning appearance, weight, shape, and size of foods in the supply chain is one important source of food waste, and that the abolition of such specifications would prevent food waste (e.g., [46,47,48,49,50]). However, thus far no research has specifically provided insights into the impact of cosmetic specifications on food waste, or on supply chain actor motivations, abilities, and opportunities to prevent suboptimal foods waste and to include them into their production systems. Our interviews revealed that abolishment of European Union specifications would not necessarily decrease waste of suboptimal foods, because actors also set cosmetic specifications in their own supply chains. The food waste resulting from cosmetic specifications depended on the type of supply chain actor, and ranged between 1% and 40% of the food production. Supply chain actor motivations, abilities, and opportunities to produce and sell suboptimal foods were mixed, and are a function of personal and organizational standards related to delivering high quality products and corporate social responsibility. They also depended on market contextual factors such as competition between suboptimal and optimal products, the pricing of suboptimal foods, production costs and logistics to produce and transport suboptimal foods, and on consumer wishes to buy such. Overall, it appeared that supply chain actors are in principle motivated to produce and sell suboptimal foods, but that especially the market context needs to change first to enable such a future perspective.

### 4.2. Take-Back Clause and Its Influence on Food Waste, Prices, and Retail Market Power

Retailer and supplier strategies and governance of the food value chain may also affect food waste. We investigated the problem of the “take-back clause” [51,52,53,54], which considers the case where the retailer is responsible for paying only for sold products; The supplier is in charge of filling up shelves and taking back unsold, close to best-before date, items. We examined the problem with semi-structured interviews with bread producers and retailers in Sweden, as bread is the main food where this agreement is widely used. Through these interviews we were able to especially understand the bread supply chain, to determine the stakeholders involved, and to investigate the extent and the effects of the take-back clause on food waste. Through our interviews of suppliers and retailers, we achieved the following:

In most cases it is the responsibility of the supplier to place fresh bread on the shelves and withdraw old (close to the best-before date) bread. Hence, the supplier operates a “circular supply chain” which is simultaneously forward (for the fresh) and reverse (for the old bread) [55,56]. The same delivery vehicle is used for both chains following the same circular route.

1. In general, not all of the unsold bread is returned to the supplier; a fraction is treated by the store itself through different channels or just ends up in the garbage of the store.
2. The supplier collects the unsold bread two to three days before the best-before date. The returned quantity differs between retail stores and between suppliers.
3. The bread inventory usually is consumed LIFO (last-in-first-out). Consumers demand freshness as prime factor when buying bread—hence they will always buy the fresher and leave behind the rest. When the suppliers need to add fresh bread, they eventually withdraw old bread 1–2 days before the best-before date. This is the trade-off for the maintenance of a certain amount of inventory at all times.
4. Bakeries consider that, to a great extent, the take-back clause is a result of the market power of the retailer. Sweden has one of the highest concentrations of retailers in Europe [57]. Retail outlets request full shelves at all time, regardless of actual demand. By paying only for what is sold, retailers push the cost of returns to the supplier. Although bakeries understand that there should always be a certain amount of bread on the shelves, they do not agree that they should bear the entire cost of unsold bread. Furthermore, it is believed that this process increases food waste because bakeries argue that they are forced to produce—and waste—more bread than they would if they had full control of how much bread should be delivered, or if retailers paid for all—both sold and unsold—bread.
5. Retailers and bakeries agree that a certain amount of waste is unavoidable. The amount of bread placed on shelves is based on forecast, not so much on actual need. Hence it is only natural to have some bread left over as waste.
6. Weather appears to be also a factor of waste, as it affects shopping behavior; unexpected weather changes may increase or decrease purchase and, therefore, influence the amount of how much is wasted.
7. The production method of bread is of importance. Bakeries which bake and freeze and then distribute frozen bread have a lower percentage of bread returns (approx. 4–5%) than those which deliver freshly baked bread (approx. 12–14%). This is because the store management is able to maintain the shelf inventory by simply taking bread out of the freezer (sometimes more than once during the day) for letting it unthaw on the shelves, hence, being always “fresh”.
8. The distribution route of the delivery vehicle of the bakery and the size of the store are also important. The last store in the route usually receives what is remaining in the lorry regardless of its actual needs, and hence often receives more than necessary. This is because the bakery agents do not want to return fresh bread to the bakery; they tend to “dump” all leftovers to the last store. Because, usually, distribution routes start with the large and end with smaller stores, it is the smaller stores that exhibit more waste in relative terms.
9. The governance of the distribution chain may affect the amount of bread waste. Some bakeries internalize the process using their own delivery trucks, while others outsource it to logistics companies. We suspect that there may be a difference between the two schemes; however, it is still unclear which structure produces more waste.

## 5. Conclusions

The approach of this project, taking account all actors in the food supply chain and also integrating the perspectives and findings of studies conducted in work packages not reported here, is what makes this a unique project with a great potential to reduce food waste by encouraging sustainable consumption of suboptimal foods. A major role in food waste is the rejection of products with imperfect physical appearance such as, e.g., a buckled cucumber, broken cookies, or food in deformed packages. Ecological and sustainability considerations might increase the pressure to develop concepts of how to present and sell suboptimal food stimuli in retail outlets:

- Choice experiments and focus groups revealed that consumers have a low tendency to purchase and consume suboptimal foods, and it is quite difficult to motivate them for doing so.
- The type of suboptimality plays a distinct role in the choice process of the consumer (as deduced from the choice experiment) and significantly impacts the influencing techniques that are necessary to motivate consumers to buy and consume suboptimal foods.
- Not only consumers are relatively pessimistic about changes of success of suboptimal food products, also supply chain actors do not perceive high chances of success for suboptimal foods. There is a mismatch between consumer needs (e.g., large discounts) to buy and consume such products, and the needs of the supply chain actor (e.g., no lower prices due to transporting problems of suboptimal products) for the selling of suboptimal foods.
- Price reduction of suboptimal foods is a powerful tool to reduce food waste at the retailer level. It should not be expected to increase food waste at home, if a high quality is assured and consumers are provided tips on usage of the items.
- Supply chain food waste reduction initiatives need good and broad collaboration as a basis, entrepreneurial spirit and drive in the organization, and a good ‘timing’ to achieve success.
- Consumers differ in their choice of suboptimal foods and food waste behavior depending on their food (waste)-related lifestyle, in particular regarding food involvement, price orientation, planning and using meals as social event, which is why policy and stakeholder actions should be targeted to different consumer segments to be more efficient.
- Intervention measures such as a mobile phone app are capable of delivering messages about food waste to consumers at home, and some consumers may benefit from using such an app to prevent wastage of suboptimal foods in the home.

## Figures and Tables

**Table 1:** Sociodemographic description of the participants of the online survey.

	Overall	Denmark	Germany	Norway	Sweden	The Netherlands
Participants	4214	848	838	851	854	823
Age (years)	44.6 ± 14.4	45.4 ± 15.4	43.9 ± 13.5	43.9 ± 14.2	45.3 ± 14.6	44.4 ± 14.2
Female (%)	51.1	51.8	51.1	50.5	50.8	51.4
With children under 18 in household (%)	31	28.9	28.3	31.5	35.8	30.3
Education (%)						
Primary education	7.8	8.5	20.5	4.2	4.7	1.2
Secondary education	22.7	11	16.1	28.4	37.2	20.5
Vocational school	27.1	24.3	37	13.4	20.6	41.1
Bachelor degree	22.5	27.7	6.2	32.1	20.5	26.1
Master degree/PhD	19.8	28.5	20.2	21.9	17	11
Household income (%)						
Less than 50% of average	20.2	19.5	25.1	18.6	14.5	23.7
Between 50% and 150% of average	57.2	52	57	57	62.9	57.1
More than 150% of average	10.8	14.5	8.6	11	13.3	6.3
Not specified	11.8	14.2	9.4	13.4	9.3	12.9
Perceived waste importance (1, not important at all; 7, very much important)	4.56 ± 1.32	4.51 ± 1.49	5.06 ± 1.23	4.32 ± 1.25	4.18 ± 1.25	4.74 ± 1.15
Do shopping/cooking (1, never; 5, always)	4.16 ± 0.83	4.23 ± 0.85	4.24 ± 0.81	4.12 ± 0.79	4.00 ± 0.81	4.18 ± 0.84

**Table 2:** Examples of the characterization of food (waste)-related consumer lifestyle segments (#1–#5) and differences in food waste and food waste-related indicators.

Food (Waste)-Related Lifestyle Dimension or Waste Indicator	Involved Socializers (#1)	Uninvolved (#2)	Price-Oriented (#3)	Well-Planning (#4)	Price-Dismissive (#5)
Meal consumption as a social event	4.89 ^a^	3.02 ^c^	2.38 ^d^	2.53 ^d^	3.26 ^b^
Self-fulfillment from cooking	5.56 ^b^	3.35 ^c^	3.49 ^c^	5.84 ^a^	5.48 ^b^
Social relations via meals	5.64 ^a^	4.24 ^c^	5.17 ^b^	5.80 ^a^	5.50 ^a^
Importance of credence attributes for quality	5.35 ^a^	3.49	3.75 ^d^	4.91 ^b^	4.61 ^c^
Norms to avoid food waste	5.85 ^a^	4.31 ^c^	5.75 ^a^	5.96 ^a^	5.29 ^b^
Cooking and culinary interest	5.66 ^a^	3.43	3.73 ^c^	5.59 ^a^	5.32 ^b^
Planning meals	3.97 ^b^	3.59 ^c^	3.18 ^d^	4.95 ^a^	3.62 ^c^
Price as criterion for shopping behavior	5.16 ^b^	3.41 ^c^	5.52 ^a^	5.55 ^a^	3.05 ^d^
Knowledge ^1^	43.6 ^a^	43.6 ^a^	41.9 ^ab^	43.3 ^a,b^	40.7 ^b^
Relative importance ^2^	5.14 ^a^	4.04 ^d^	4.51 ^b^	4.87 ^a,b^	4.41 ^b^
Tendency to choose‚ optimal‘ at home	3.98 ^a^	4.06 ^a^	3.12 ^bc^	2.97 ^c^	3.48 ^b^
Self-reported food waste at home ^3^	16.0 ^a^	18.0 ^a^	10.0 ^bc^	8.0 ^c^	11.0 ^b^

Respondents’ assessment measured on a 7-point Likert disagree/agree scale. Statistical test: One-way ANOVA with post-hoc Games–Howell test. Significant mean differences in group comparison in the post-hoc test (with *p* < 0.001) are indicated by different superscript letters. For all ANOVA’s: *p* < 0.001. ^1^ estimated world food waste in percentage; ^2^ of the topic of food waste vs. stabilizing the economy, ^3^ percentage mean across categories.
